# MAMI: a birth cohort focused on maternal-infant microbiota during early life

**DOI:** 10.1186/s12887-019-1502-y

**Published:** 2019-05-03

**Authors:** Izaskun García-Mantrana, Cristina Alcántara, Marta Selma-Royo, Alba Boix-Amorós, Majda Dzidic, Jose Gimeno-Alcañiz, Isabel Úbeda-Sansano, Ignacio Sorribes-Monrabal, Ramón Escuriet, Fernando Gil-Raga, Anna Parra-Llorca, Cecilia Martínez-Costa, María Carmen Collado, on behalf of MAMI team, Maria Carmen Collado, Izaskun García-Mantrana, Cristina Alcántara, Jose Gimeno-Alcañiz, Marta Selma-Royo, Alba Boix-Amorós, Majda Dzidic, Christine Baüerl, Eva Villoldo, Carlos Zafra, Laura Olivares, Gaspar Pérez-Martínez, Alex Mira, Maria Desamparados Ferrer, Jacobo Martínez Santamaria, Andrea Ahicart, Máximo Vento, Anna Parra-Llorca, María Gormaz, María Cernada, Cecilia Martínez-Costa, Bibiana Bertúa-Ríos, Beatriz Padilla, Elena Crehuá-Gaudiza, Alba Peretó-Moll, Amparo Rodríguez García, Maria Dolores Soler Rico, Fernando Gil-Raga, Nuria Bixquert Martínez, Isabel Úbeda-Sansano, Irene Rausell Segarra, Jose Luis Tortajada Soriano, Ignacio Sorribes-Monrabal, Luis C. Blesa-Baviera, Amelia Peris Vidal, Llanos Madrigal Hornos, Ramón Escuriet, Teresa Gonzalo del Moral, Pepi Domínguez Cano, Marga Franch i Ferrer, Concha Delgado, Adela Atero

**Affiliations:** 1 Department of Biotechnology, Institute of Agrochemistry and Food Technology-National Research Council (IATA-CSIC), Av. Agustin Escardino 7, 46980 Valencia, Spain; 2 Health care center La Eliana, Valencia, Spain; 3 Health care center Serreria II, Valencia, Spain; 4 División de Innovación y Cartera de Servicios Sanitarios, Area de Atención Sanitaria y Servicios de Salud. Generalitat de Catalunya, Centre for Research in Health and Economics, Universidad Pompeu Fabra, Barcelona, Spain; 5 Department of Obstetrics & Gynaecology Hospital de MANISES, Valencia, Spain; 6 Health Research Institute La Fe, Neonatal Research Group, Spain and University and Polytechnic Hospital La Fe, Division of Neonatology, Valencia, Spain; 7 Department of Pediatrics, Hospital Clínico Universitario, University of Valencia, Valencia, Spain

**Keywords:** Cohort study, Microbiota, Diet, Lactation, Neonates, Meconium, Early nutrition, Growth, Breast milk, Antibiotics, Birth

## Abstract

**Background:**

Early microbial colonization is a relevant aspect in human health. Altered microbial colonization patterns have been linked to an increased risk of non-communicable diseases (NCDs). Advances in understanding host-microbe interactions highlight the pivotal role of maternal microbiota on infant health programming. This birth cohort is aimed to characterize the maternal microbes transferred to neonates during the first 1000 days of life, as well as to identify the potential host and environmental factors, such as gestational age, mode of delivery, maternal/infant diet, and exposure to antibiotics, which affect early microbial colonization.

**Methods:**

MAMI is a prospective mother-infant birth cohort in the Spanish-Mediterranean area. Mothers were enrolled at the end of pregnancy and families were follow-up during the first years of life. Maternal-infant biological samples were collected at several time points from birth to 24 months of life. Clinical and anthropometric characteristics and dietary information is available. Specific qPCR and 16S rRNA gene sequencing as well as short chain fatty acid (SCFAs) profile would be obtained. Multivariable models will be used to identy associations between microbiota and clinical and anthropometric data controlling for confounders.

MAMI would contribute to a better understanding of the interaction between diet, microbiota and host response in early life health programming, enabling new applications in the field of personalized nutrition and medicine.

**Trial registration:**

The study is registered on the ClinicalTrial.gov platform NCT03552939. (June 12, 2018).

## Background

Scientific evidence has shown the relevance of gut microbiota for optimal health, due to its impact on physiology, metabolism, and immune response. Microbial dysbiosis, alterations in the gut microbiota composition and activity, has been related to higher risk and susceptibility to develop non-communicable disorders (NCDs) such as obesity, allergies, inflammatory bowel disease, diabetes, and other inflammation-related problems [1]. There is increasing evidence revealing the key role of early microbial development in human health [2]. The first 1000 days of life, from conception to two years of age, is considered a window of opportunity for health programming [3]. Early colonization of the infant gut takes place during this critical period and is related to the immune, metabolic, and intestinal homeostasis development. This time frame represents a critical moment in setting the stage for future health. Maternal microbiota represents the most important source of neonatal microbes. Gut microbiota dysbiosis would reflect the impact of specific perinatal factors and adverse maternal environmental exposures during this period of time [4]. It is well-known that mode of delivery and lactation shape the neonatal gut microbiota. C-section Newborns are not exposed to the vaginal and gut microbial environment, avoiding this first and initial exposure. Those neonates had a delayed gut colonization of *Bacteroides* and *Bifidobacterium* groups, along with a dysbiotic microbial profile and lower diversity than the microbial profile observed in vaginally born infants [5–7].

However, limited data is available on the impact of perinatal factors, such as gestational age, antibiotics exposure, diet, specific nutritional compounds, and lifestyle on maternal microbiota. Then, shifts in the maternal microbiome could be vertically transmitted to the neonate, at birth and also, during lactation, promoting an altered microbial inoculum that may influence infant development with short- and long-term health consequences. Human breast milk (HM) is considered the key postnatal link between mother and infant, and it is recognised as the gold standard for infant nutrition, as recommend by World Health Organization (WHO). HM “constantly” change in composition according to the neonatal requirements [8, 9], HM contains nutrients and other bioactive components as well as several immune-related substances together with microbes and oligossacharides (HMO) that directly influence the neonatal microbiota and immune system development [10–14].

Perinatal and environmental factors, including mode of delivery, gestational age and weight status as well as diet and life-style, would affect the microbial and non-microbial components of breastmilk, impacting infant gut and health development. However, information in this regard is lacking, and the relationship remains unclear. In this scenario, it is needed to increase our understanding in host-microbe interactions mainly in the first 1000 days as in this period maternal microbiota exerts a pivotal role on infant health programming. It is not yet known to what extent the specific composition of the maternal microbiome influences the infants’ microbial colonization, nor how environmental factors could affect composition in the mother and subsequently, in the infant. The overall objective of MAMI cohort is to analyze early microbial exposition focusing on maternal microbiota, including breast milk, shaping the neonatal oral and intestinal microbiota development and immune system maturation. For that reason, it is very important to determine the impact of perinatal and environmental factors on maternal-neonatal microbiota during early life.

The specific aims of this birth cohort study are:

- To understand how the maternal microbiome is influenced by environmental factors during the perinatal period.
- To characterize the microbial core and bioactive compounds transmitted to the offspring via breastfeeding and identify their role in the gut microbiota development.
- To identify and study the interactions among microbiota and breast milk bioactive compounds and their influence on intestinal, metabolic, and immune system development in infants.

## Methods

### Study design and recruitment

MAMI study (“MAternal MIcrobes”) is birth longitudinal prospective observational cohort study that includes mother-infant pairs followed during first 24 month of life in the Mediterranean area of Spain.

#### Recruitment

Women were enrolled either during pregnancy and/or within 1 week after birth. Recruitment initially aimed to reach women at the end of the pregnancy via gynaecologists and midwives who provided basic information about the study and also, in the primary health centers and hospitals. The MAMI cohort study was disseminated with flyers, posters and also, information sheets at primary health-care centers and hospitals, and also through verbal information with physicians, midwives, other team members, and also, in social networks (Facebook, webpage and twitter).

#### Time/Duration

Recruitment started in 2015 and the follow-up was completed early 2019. Written informed consent was obtained from all the participants.

#### Protocol and Ethics

The study protocol with the registration number 2015/0024 was approved by Hospital ethics committees (HECs) involved in MAMI (Hospital Universitario y Politécnico La Fe, Hospital Clinico Universitario de Valencia, Atención Primaria Comunidad Valencia, and CEIC-Parc de Salut MAR), as well as by the local ethics committee of Atención Primaria- Generalitat Valenciana (CEIC-APCV). The study is registered on the *ClinicalTrial.gov* platform, with the registration number NCT03552939.

### Study population

A total of 250 mother-infant pairs were initially recruited. The inclusion criteria required the mother to be older than 18 years old, healthy pregnancy and to be able to understand written and spoken Spanish. The exclusion criteria were non-compliance with any of the inclusion criteria, use of medications or drugs, suffering chorioamnionitis or health complications during the gestational period, as well as experiencing any chronic disease or taking medication for any chronic pathology such as diabetes or pre-gestational thyroid problems. Participants were required to live in the Mediterranean area (Spain) to ensure lifestyle homogeneity and environmental exposures, avoiding confounding factors that could impact the relationship between microbiota and variables of interest.

### Sample collection timeline

**Fig. 1:**
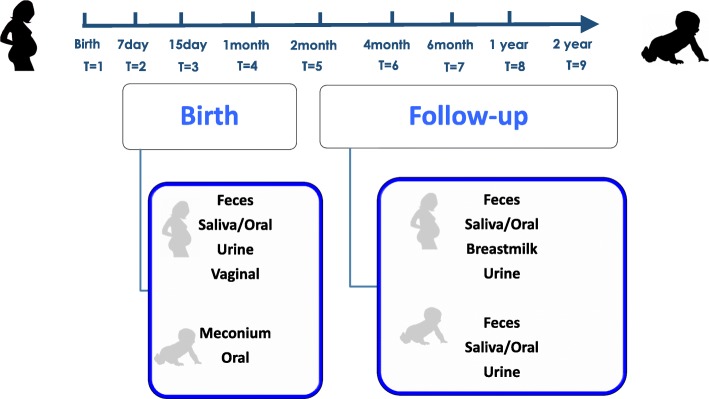
Flow chart of sampling procedure

The mother-infant cohort was monitored from birth until two years of life. Biological samples were collected throughout this period at different time-points, as described in Fig. 1. Participants were provided with verbal and written information at any time when samples were collected.

At the designated times, non-invasive faecal, oral, and urine samples were collected from mothers and infants. Vaginal swabs were collected just before birth. In addition, breastmilk samples were also collected during lactation period and also, when samples were available. All health professional collaborators and participating mothers were provided verbal and written instructions about sample collection.

Faeces, urine and breastmilk were collected in sterile containers of different sizes and shapes. For the standardized milk collection, breast skin was cleaned with 0.5% chlorhexidine solution and first drops were discarded. Morning collection was recommendable. Then, breast milk was collected by use of a sterile pumper in sterile bottles to normalize the milk collection. Finally, breast milk samples were sent to biobank and then, aliquoted and stored at − 80 °C until further analysis. Availble breastmilk samples were collected from mothers following breastfeeding practices at the designated time-points. Once, the mothers decide to stop breastfeeding, there would be not available milk sample. Oral sampling consisted of simple swabbing of maternal and infant inner part of the cheek with sterile oral swabs. For the morning saliva collection, the Salivette® was used. All samples were sent to biobank, and then, biological samples were managed and stored in sterile cryovials under specific standardized protocols at “Biobanco para la Investigación Biomédica y en Salud Pública de la Comunidad Valenciana (IBSP-CV)”. Once all cohort samples were collected and placed in biobank, aliquots were shipped and centralized at IATA-CSIC for the analysis.

### Data collection

Perinatal information was recorded while samples were collected during each visit. Such perinatal information included:

- Mode of delivery and place of delivery.
- Group B *Streptococcus* status.
- Exposure to antibiotics.
- Anthropometric measurements.
- Apgar test score.
- Maternal age.
- Gestational age.
- Maternal weight before gestation and weight gain over the pregnancy.
- Smoking status or maternal smoking exposure and alcohol consumption during pregnancy.
- Atopic disease, allergies, and other possible disorders.
- Exclusive and/or partially-breastfeeding practices

These data were obtained from clinical records collected by clinicians at hospitals and primary health centres. Information related to pregnancy and delivery was retrieved from the intrapartum medical record by the medical staff and gynecology team. Infant health outcome measurement was recorded and provided by the pediatrician’s team.

To determine dietary intake during pregnancy and the follow-up period, each participant was asked to answer a comprehensive 140-item validated food frequency questionnaire (FFQ) [15]. In all cases, FFQ information registered by participants were validated by use of a three-day food record questionnaire for the intake of dietary nutrients. In addition, a 14-item, PREDIMED (PREvención con DIeta MEDiterránea) validated test was also used to appraise adherence of participants to the Mediterranean diet [16]. Diet questionnaires and supplement intake records were collected by a nutritionist within 24 h after birth, representing maternal diet during gestations, and then, during the follow-up period (at 12 and 24 month). Data recorded from the infant included anthropometric measurements, type of feeding, diet, antibiotic exposure, and health status at each visit throughout study completion.

### Ethical procedure and confidentiality

Several steps were taken to maintain the confidentiality of subjects. No identifying information has been noted in the metadata. All participants have been assigned an anonymous code. Personal identification data has not and will not be made public at any time. Subjects may withdraw consent for participating in the study at any time.

### Primary outcome and covariate assessment

**Fig. 2:**
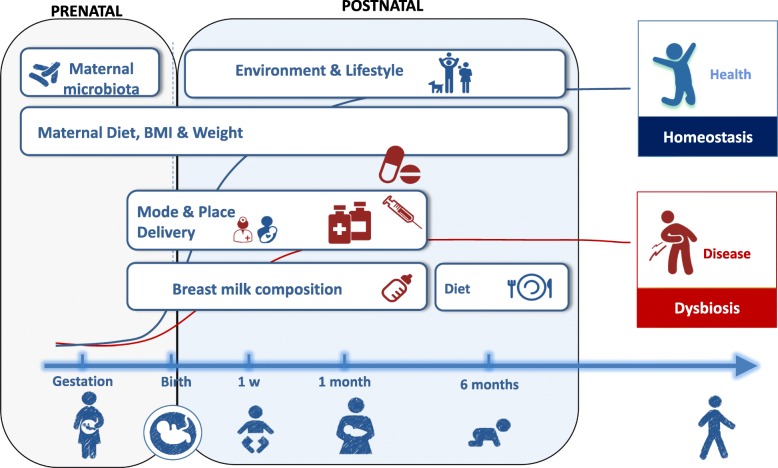
Influencing factors on microbiota development during early life

The primary outcome is to characterize the microbial community, diversity, and activity in mother-infant interphase by use of both, culture-dependent microbiological techniques and culture-independent methodologies based on PCR, from stool, oral, and breastmilk samples collected at several points during the first two years of infant life. In addition, we investigate potential confounding factors or effects that would affect the mother-infant microbial transmission during first years of life (Fig. 2), including:

- The effect of maternal diet on microbiome composition during gestation and 12 months after childbirth.
- The effect of overweight and obesity as well as an excessive weight gain over the pregnancy, along with maternal weight loss from birth until 12 months postpartum.
- The influence of mode of delivery (vaginal, elective, and non-elective C-section) and place of delivery (inborn or outborn) on microbiota development.
- The effect of antibiotic exposure during gestation and the perinatal period on microbial composition.
- The infant growth and development by use of WHO standards
- The effect of the infant diet on gut microbiota from birth to study completion. We will study the effect of exclusive breastfeeding compared to formula feeding (type of formula and also, the presence of probiotics, prebiotics and HMO were recorded), the inclusion of formula after a breastfeeding period, and finally, complementary food introduction. Besides microbial composition, we also will analyse the non-microbial components and the immune-related substances present in breastmilk in breastfeeding mothers and their role in immune system development and health.

Other covariates included in the study were: maternal age, gestational diabetes, medications and infections during gestation and first degree relative history of atopic disease, allergies or other problems. Furthermore, maternal-neonatal perinatal information birth weight, length at birth, sex, antibiotic, corticoids, and analgesia use were also collected. During the follow-up visits, data on infant growth, respiratory and gastrointestinal infections, use of antibiotic and antifungal, medications as well as changes in food pattern were collected by clinicians and recorded in specific clinical forms at hospital and primary health centers. Those information was accessible for MAMI team clinician members.

We aim to investigate the influence of these factors and covariates on the maternal microbiome and microbiota development in the offspring, along with their relationship with infant growth during early infancy. We would also explore the influence of microbial shifts on immune system maturation and the specific health outcomes, such as obesity, allergies, and respiratory infections.

### Microbiota analysis

#### DNA extraction

DNA extraction from the different samples are obtained by use of commercial kit, the Master-Pure DNA Extraction Kit (Epicentre, Madison, WI, USA), with an enzymatic treatment with lysozyme and mutanolysin and a mecanical step of cell disruption with glass beads, followed by bead beater to enhance DNA recovery and concentration as previously described protocol [17].

#### Microbiota composition by qPCR

PCR primers targeted to total bacteria and specific bacterial group as *Bifidobacterium* group and species including *B. longum*, *B. bifidum*, *B. breve*, *Lactobacillus*, *Enterococcus*, *Bacteroides*, *Enterococcus*, *Staphylococcus* and *Streptococcus* groups, Enterobacteriaceae family, *Akkermansia muciniphila, Faecalibacterium, Fusobacterium* are conducted as previously described [18].

qPCR are performed in LightCycler® 480 Real-Time PCR System (Roche®) by use of SYBR® Green PCR Master Mix (Roche®). A melting curve analysis is made at the end of the PCR and bacterial concentrations are calculated by comparing the Ct values from standard curves.

#### Microbiota composition by 16S rRNA gene amplicon sequencing

DNA is purified by use of DNA Purification Kit (Macherey-Nagel, Duren, Germany) according to manufacturer’s instructions. DNA concentration is measured using Qubit® 2.0 Fluorometer (Life Technology, Carlsbad, CA, USA) and normalized to 5 ng/μl for amplicon libraries. Specific 16S rRNA gene (V3-V4 region) is amplified by PCR using Illumina adapter overhang nucleotide sequences following Illumina protocols. After amplification, the mutiplexing step is performed using Nextera XT Index Kit. 16S amplicons are confirmed and checked with a Bioanalyzer DNA 1000 chip and libraries are sequenced using a 2x300pb paired-end run (MiSeq Reagent kit v3) on a MiSeq-Illumina platform (FISABIO sequencing service, Valencia, Spain). To rule out and control for possible reagent contamination, DNA-reagents and DNA-extraction and PCR amplification are also included and sequenced as controls.

#### Microbial activity by SCFA analysis

Short chain fatty acids (SCFA) are identified by high pressure liquid chromatographic methods (Jasco Corporation, Japan) as described [19]. Stool samples are homogenized and centrifuged at low speed, the supernants are filtered and mixed with phosphoric acid 0.1% as a mobile phase. Then, mixture is injected into the HPLC system, comprised of a column Rezex™ ROA-Organic Acid H+ (8%), LC Column 300 × 7.8 mm, (Phenomenex, USA) and a UV detector at 210 nm.

### HMO analysis

HMO identification in milk samples are carried out by use of HPAEC-PAD (High performance anion exchange chromatography with pulsed amperometric detection) analysis via a Dionex BioLC system, as described [20, 21]. Breast milk samples are centrifuged to remove the fat, whey milk is filtered and injected into the Dionex system, comprised of a column Dionex CarboPac PA100, (Thermo Scientific) at 30 °C, and an electrochemical detector. NaOH 1 M and sodium acetate 1 M was used as a mobile phase.

### Immune markers profiling analysis

Immune parameters and cytokine profiles from non-invasive mother-infant samples (mainly in breast milk samples but also, faeces and oral samples) are analysed by use of Luminex-approach and also, ELISA determination. Concentrations of the specific cytokines and the chemokines, macrophage migration inhibitory factor, monocyte chemo-attractant protein-1, macrophage inflammatory protein-1-α, eotaxin, and also, immunoglobulins among others, will be determined.

### Power calculation and sample size

MAMI initially recruit for the study 250 pregnant women, in order to complete the study with a minimum of 100 mother-infant pairs. Building a birth cohort that involves a long follow-up period, large sample size, and frequent data collection is always challenging. To minimize losses during follow-up, we developed strategies to keep participants informed of our advances, developing diaries, meetings, talks, and creating a web page to keep participants informed. These techniques were used as a reminder of the study’s benefits to the community and field of child health. It was our intention to raise the participating families’ awareness of the contributions the study would make towards understanding how early risk factors could affect future health through the microbiome. Due to this effort, the recruitment and the collection of samples and data is attainable, and we maintained a very low attrition rate. It is for that reason that we decided to continue with the same strategies moving forward. The second year of follow-up has been recently completed.

### Statistical analysis and data integration

Statistical analyses of the qPCR data would include parametric and non-parametric tests according to the data distribution. In case of non-normal distribution, data is Log transformed and parametric tests as T-test are performed. Parametric repeated measures ANOVA test was used to compare microbial groups along time (paired samples). The χ-square test is applied to establish differences in bacterial prevalence between the studied groups. A *P* < 0.05 is considered statistically significant.

Statistical analyses of the 16S rRNA gene sequence data were performed with QIIME pipeline tools. Chimeric sequences and sequences not assigned to Bacteria domain level, also, those sequences classified as cyanobacteria and chloroplasts, likely represent ingested plant material, are removed from the dataset. Alpha diversity (Chao1 and Shannon indexes) and beta diversity based on UNIFRAC distance (phylogenetic) and Bray Curtis distance (non-phylogenetic) are obtained. Permutational multivariate analysis of variance-PERMANOVA- with 999 permutations is used to test significance between microbiota in different groups. Principal coordinate analysis (PCoA) plots with phylogenetic and non-phylogenetic distances were produced. To test for statistically significant differences in taxonomic richness, diversity measures, bacterial load, and bacterial abundances, appropriate tests, such as one-way ANOVA with corrections (Dunn’s multiple comparisons) and Tukey HSD test as a post-hoc test are used. For each analysis, a false discovery rate (FDR) correction would be applied to adjust *P* values. R packages and Calypso software (http://cgenome.net/calypso/) are used for statistical analysis and multivariate testing. Furthermore, inferred metagenomics Functional analysis, the PICRUSt (Phylogenetic Investigation of Communities by Reconstruction of Unobserved States) approach would be used as described [22]. Linear discriminant analysis effect size (LEfSe) [23] is used to identify specific microbial features and specific funcions (KEGG pathways) as biomarkers between groups of samples. Multivariable models will be used to identy associations between microbiota and clinical and anthropometric data controlling for confounders.

## Discussion

### Loyalty and study strength and limitations

We started with a recruitment of 250 pregnant women in order to complete the study with a minimum of 100 mother-infant pairs. Clinical record and antoprometric data were collected from all participants however, some biological samples were not collected and some families did not participate in all follow-up. The drop out rate taking into account the families not involved in the follow-up was around 10%. There are several strengths and also, some limitations to our study. The biological samples, including faeces, urine, saliva, and breast milk samples, were obtained by use of non-invasive procedures, and their collection should cause no more than minimal discomfort.

One of the most outstanding strength of this study is the information obtained about participants during the first year of life, a pivotal period for health programming. Contact with hospitals and community health centres greatly aided to reduce the rate of missing data. Promoting good relationships with participants could facilitate extending the life of the cohort. Nevertheless, the follow-up period has presented challenges. We have to acknowledge the great effort and implication of the participants in this study. Currently, we are working on improving cohort retention.

Another key factor of this study is the multidisciplinary staff involved. The staff members are specialized, persistent, and collaborate well in accomplishing their retention goals. However, we also find some limitations in our study, such as the brief duration of the follow-up period and the lack of data regarding other exposures (e.g. pollution, chemicals, etc). We must also account for the limitations of the food frequency questionnaires, including errors and biases in food quantity estimations.

### Implications

The potential benefits of this study will provide further understanding of the colonization and development of the human microbiome during early life. Furthermore, because of the important role microbiota plays on metabolic and immunological programming of a human being, this research may help to promote new preventive and therapeutic strategies that will result in long-term health benefits for a large proportion of population currently suffering from diseases related to dysbiosis, such as the development of new dietary strategies and therapies based on microbial modulation. It will also provide new tools to promote medical practices that affect microbiota development, such as infant-mother diet and the use of antibiotics.

## Abbreviations

BMI: Body mass index
HMO: Human milk oligosaccharides
KEGG: Kyoto encyclopedia of genes and genomes
SCFA: Short chain fatty acids
